# Supplementary material for: Effect of stimulation time on the expression of human macrophage polarization markers
Source: PLoS One. 2022 Mar 14;17(3):e0265196. doi: 10.1371/journal.pone.0265196 (PMC8920204; doi:10.1371/journal.pone.0265196)
Supplement: S1 Table — Expression of the indicated markers at the time points shown were compared by repeated measures ANOVA, * p < 0.05; ** p < 0.01; *** p < 0.001. ns, not significant; US, unstimulated. (PDF) [file pone.0265196.s001.pdf]

M1							M2a						M2c			
	CXCL9	CXCL10	TNF	IL-1β	IL-12	IDO1		MRC1	TGM2	CCL17	CCL22	IL-10		IL-10	CD163	TGFβ
US vs. 4h	**	***	***	**	**	*	US vs. 4h	***	***	*	***	***	US vs. 4h	***	***	ns
US vs. 8h	***	***	***	**	***	**	US vs. 8h	***	**	*	***	***	US vs. 8h	***	**	ns
US vs. 12h	**	**	***	***	***	***	US vs. 12h	***	**	*	**	***	US vs. 12h	ns	***	**
US vs. 24h	*	**	***	*	ns	ns	US vs. 24h	***	***	*	***	***	US vs. 24h	**	***	ns
US vs. 48h	**	***	***	**	***	**	US vs. 48h	***	**	*	**	***	US vs. 48h	***	***	***
US vs. 72h	*	**	***	*	*	***	US vs. 72h	***	**	*	***	***	US vs. 72h	***	***	***
4h vs. 8h	**	***	***	***	***	**	4h vs. 8h	ns	ns	ns	***	***	4h vs. 8h	**	ns	ns
4h vs. 12h	**	***	***	*	***	**	4h vs. 12h	*	ns	*	**	**	4h vs. 12h	***	**	*
4h vs. 24h	**	***	***	ns	**	ns	4h vs. 24h	ns	ns	**	***	***	4h vs. 24h	***	ns	ns
4h vs. 48h	**	***	***	ns	ns	**	4h vs. 48h	*	ns	ns	**	***	4h vs. 48h	***	**	*
4h vs. 72h	**	***	***	***	ns	***	4h vs. 72h	***	*	**	***	***	4h vs. 72h	**	***	ns
8h vs. 12h	***	**	***	ns	ns	ns	8h vs. 12h	ns	*	*	*	ns	8h vs. 12h	***	ns	ns
8h vs. 24h	***	***	***	ns	**	ns	8h vs. 24h	***	*	*	***	***	8h vs. 24h	**	ns	ns
8h vs. 48h	**	***	***	ns	***	**	8h vs. 48h	*	ns	ns	**	***	8h vs. 48h	ns	*	**
8h vs. 72h	***	***	***	*	**	***	8h vs. 72h	***	***	*	**	***	8h vs. 72h	ns	*	ns
12h vs. 24h	ns	ns	**	ns	*	ns	12h vs. 24h	***	ns	ns	***	***	12h vs. 24h	**	**	ns
12h vs. 48h	ns	**	ns	ns	**	**	12h vs. 48h	*	ns	*	**	***	12h vs. 48h	***	***	*
12h vs. 72h	**	**	*	ns	**	***	12h vs. 72h	***	**	ns	**	***	12h vs. 72h	*	***	ns
24h vs. 48h	ns	**	*	ns	**	*	24h vs. 48h	***	ns	**	*	***	24h vs. 48h	**	***	*
24h vs. 72h	*	**	**	ns	*	***	24h vs. 72h	***	**	ns	**	ns	24h vs. 72h	ns	*	ns
48h vs. 72h	*	*	***	*	ns	ns	48h vs. 72h	***	**	**	ns	***	48h vs. 72h	ns	ns	ns
