# Supplementary material for: Effect of stimulation time on the expression of human macrophage polarization markers
Source: PLoS One. 2022 Mar 14;17(3):e0265196. doi: 10.1371/journal.pone.0265196 (PMC8920204; doi:10.1371/journal.pone.0265196)
Supplement: S3 Table — Expression of the indicated markers at the time points shown were compared by repeated measures ANOVA, * p < 0.05; ** p < 0.01; *** p < 0.001. ns, not significant; US, unstimulated. (PDF) [file pone.0265196.s003.pdf]

M1				M2a		M2c	
	IL12p70	TNF	IL1β		IL10		TGFβ
US vs. 4h	***	***	ns	US vs. 4h	**	US vs. 4h	ns
US vs. 8h	***	***	*	US vs. 8h	**	US vs. 8h	ns
US vs. 12h	***	***	*	US vs. 12h	**	US vs. 12h	-
US vs. 24h	***	***	*	US vs. 24h	ns	US vs. 24h	ns
US vs. 48h	***	***	ns	US vs. 48h	***	US vs. 48h	ns
US vs. 72h	***	**	ns	US vs. 72h	***	US vs. 72h	**
4h vs. 8h	***	*	ns	4h vs. 8h	-	4h vs. 8h	ns
4h vs. 12h	***	*	*	4h vs. 12h	-	4h vs. 12h	ns
4h vs. 24h	***	**	*	4h vs. 24h	ns	4h vs. 24h	ns
4h vs. 48h	***	***	ns	4h vs. 48h	***	4h vs. 48h	ns
4h vs. 72h	***	***	ns	4h vs. 72h	***	4h vs. 72h	**
8h vs. 12h	***	ns	*	8h vs. 12h	-	8h vs. 12h	ns
8h vs. 24h	*	***	ns	8h vs. 24h	ns	8h vs. 24h	ns
8h vs. 48h	ns	***	ns	8h vs. 48h	***	8h vs. 48h	ns
8h vs. 72h	ns	***	ns	8h vs. 72h	***	8h vs. 72h	**
12h vs. 24h	**	**	ns	12h vs. 24h	ns	12h vs. 24h	ns
12h vs. 48h	*	***	ns	12h vs. 48h	***	12h vs. 48h	ns
12h vs. 72h	*	***	*	12h vs. 72h	***	12h vs. 72h	**
24h vs. 48h	**	***	ns	24h vs. 48h	***	24h vs. 48h	ns
24h vs. 72h	*	***	ns	24h vs. 72h	***	24h vs. 72h	**
48h vs. 72h	ns	ns	ns	48h vs. 72h	ns	48h vs. 72h	**
