# Supplementary figures and images for: Effect of stimulation time on the expression of human macrophage polarization markers
Source: PLoS One. 2022 Mar 14;17(3):e0265196. doi: 10.1371/journal.pone.0265196 (PMC8920204; doi:10.1371/journal.pone.0265196)

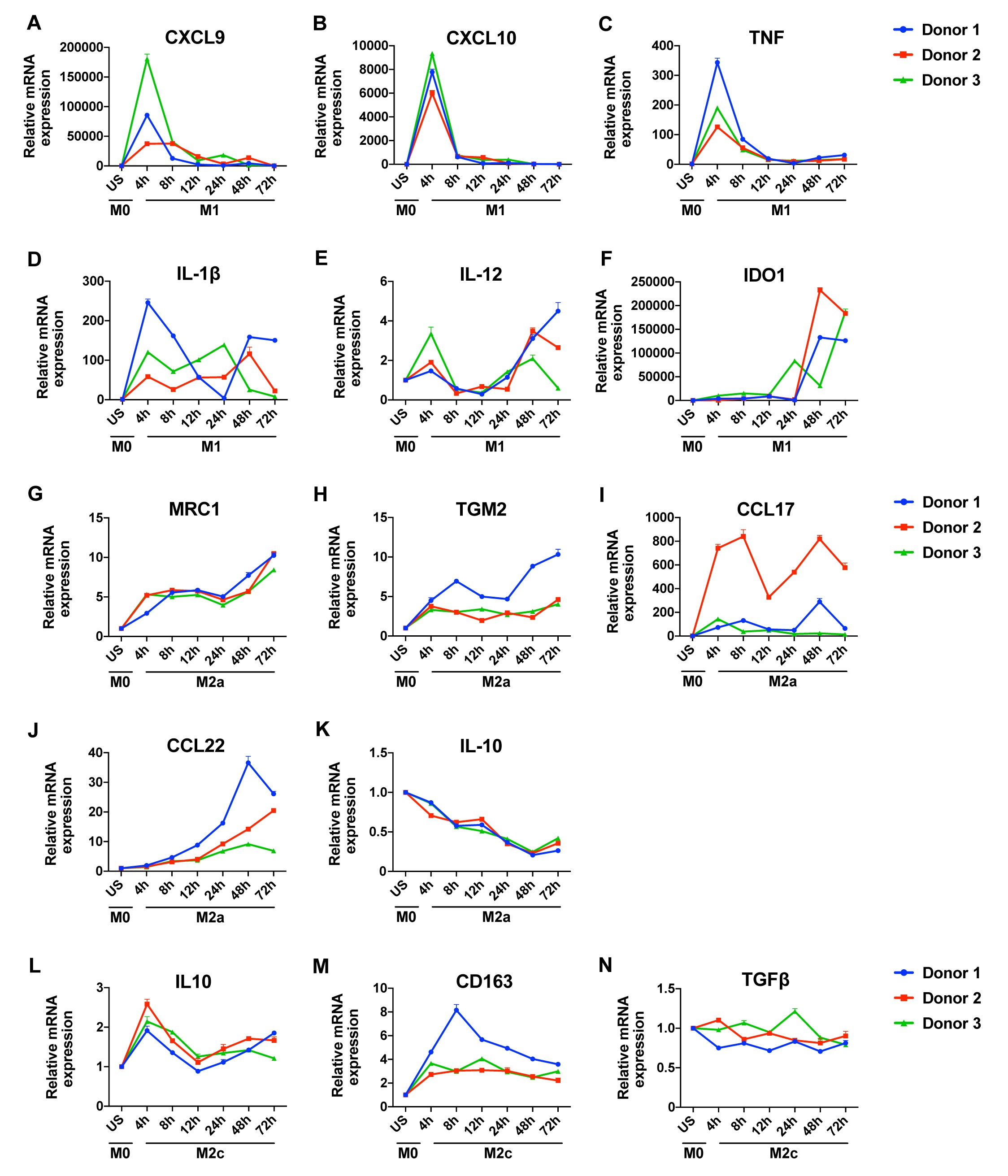

Supplement: S1 Fig — (TIF) [file pone.0265196.s004.tif]

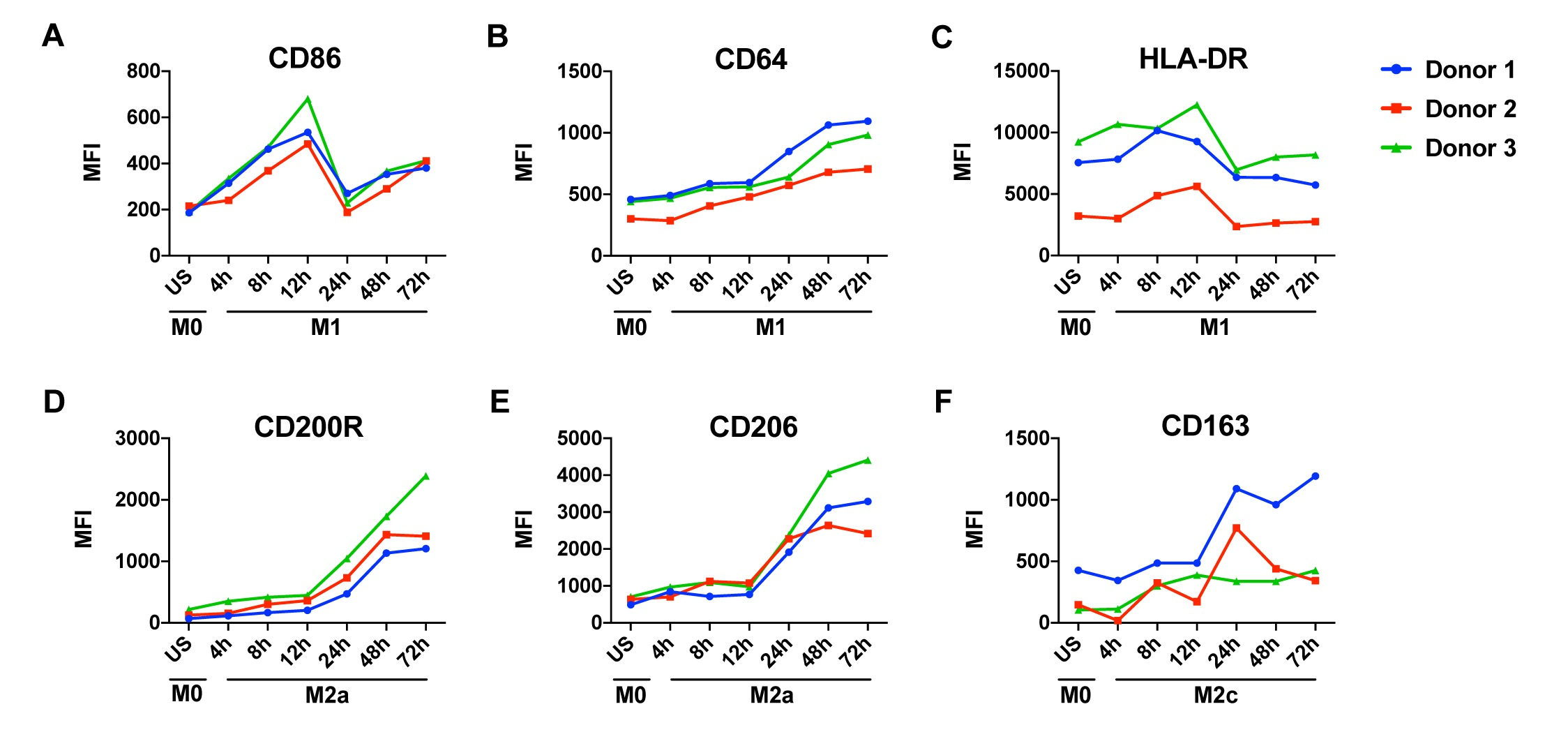

Supplement: S2 Fig — (TIF) [file pone.0265196.s005.tif]

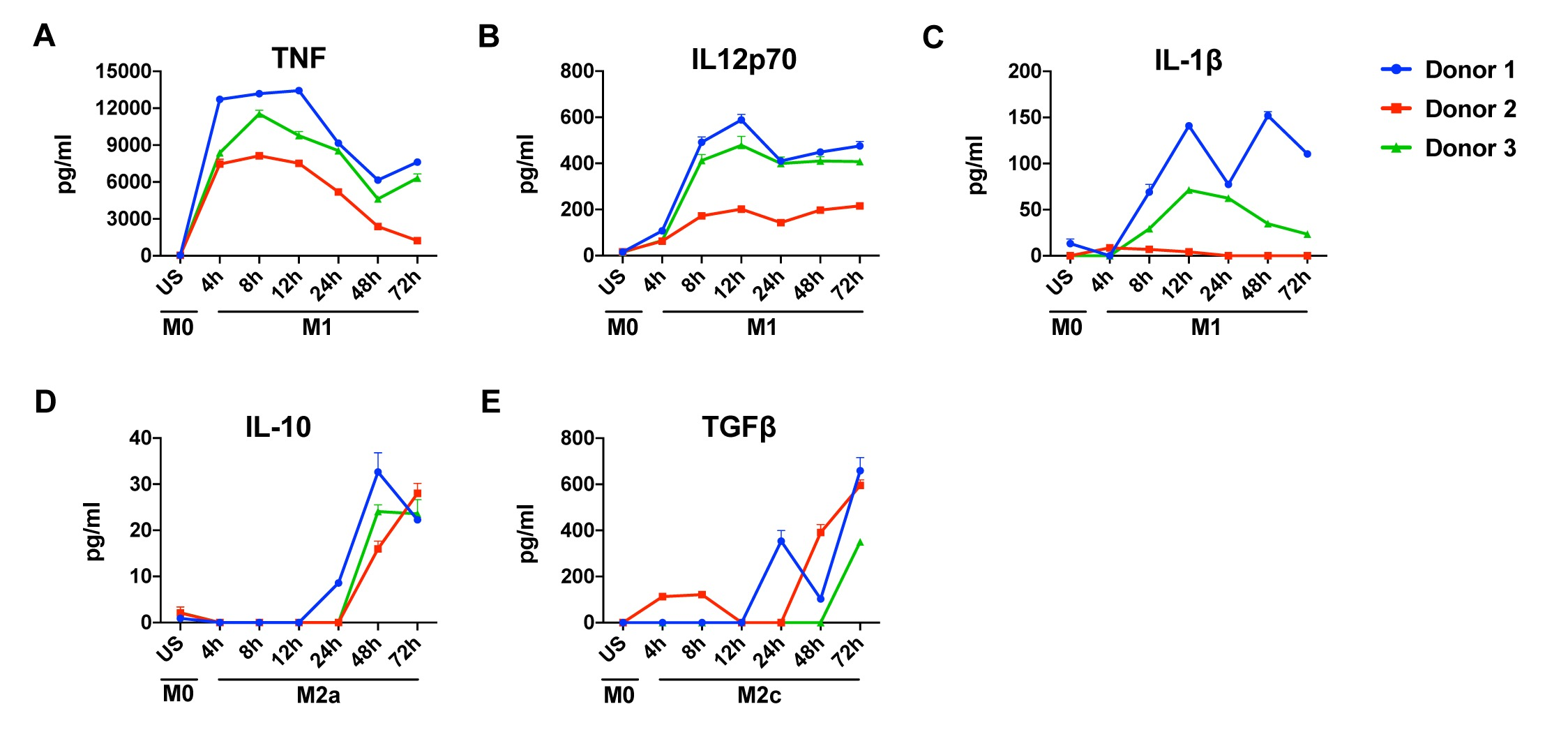

Supplement: S3 Fig — (TIF) [file pone.0265196.s006.tif]

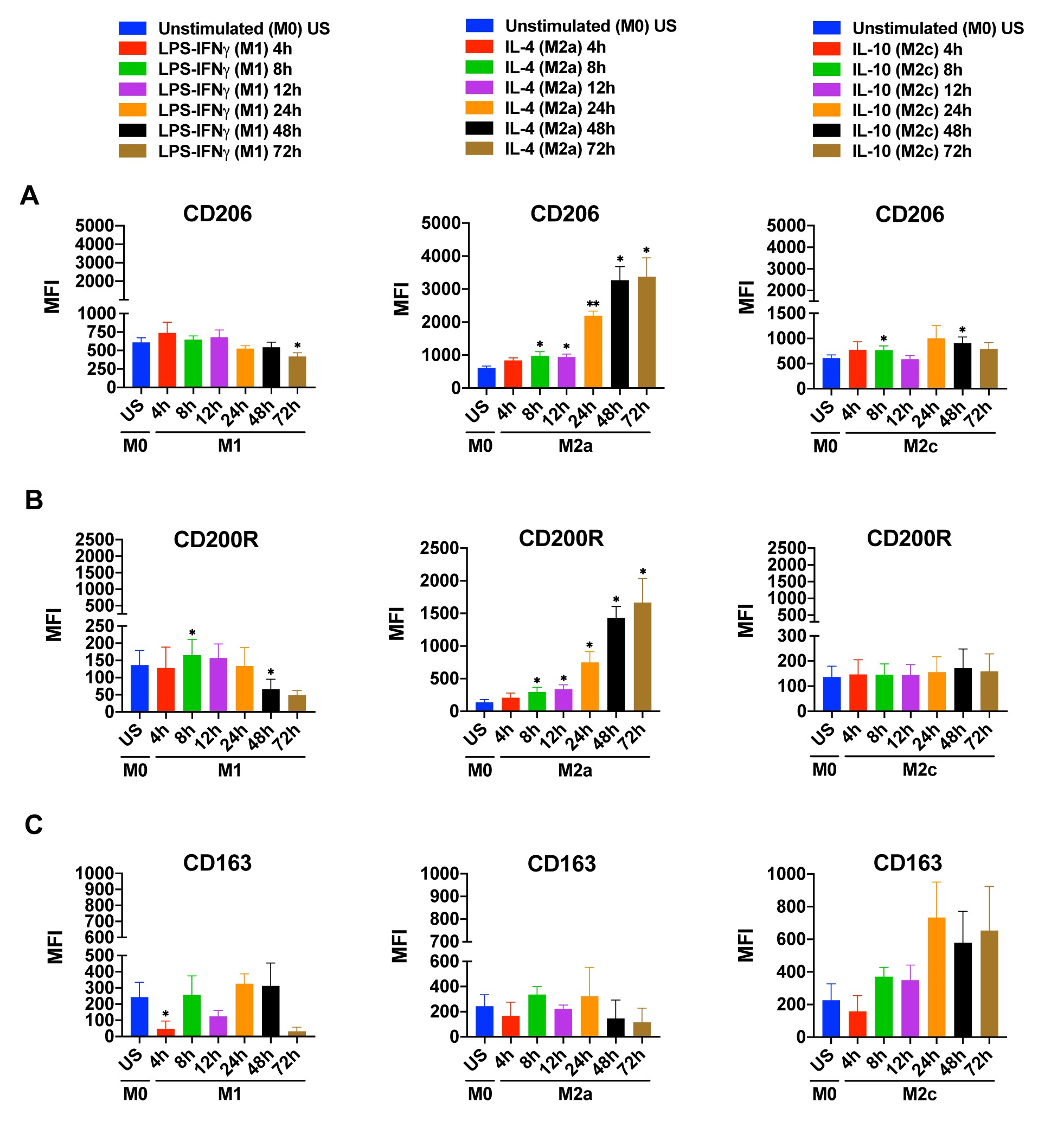

Supplement: S4 Fig — Summary data shown are mean ± SEM of biological replicates of 3 independent donors. Polarized macrophages (M1, M2a, or M2c) at all time points were compared with unstimulated (US) M0 macrophages. Statistical analyses were performed with repeated measures ANOVA, * p < 0.05; ** p < 0.01; *** p < 0.001. (TIF) [file pone.0265196.s007.tif]
