# Supplementary material for: Effect of stimulation time on the expression of human macrophage polarization markers
Source: PLoS One. 2022 Mar 14;17(3):e0265196. doi: 10.1371/journal.pone.0265196 (PMC8920204; doi:10.1371/journal.pone.0265196)
Supplement: Attachment — Submitted filename: Review PlosOne_macro time.docx [file pone.0265196.s008.docx]

**Review 30/09/2021**

Article from Purcu et al, PLOS One

This article was designed by the authors to identify the best time of activation (from 4h to 72h) to characterize specific markers of human monocyte-derived macrophages polarized into M1, M2a and M2c by using RT-PCR for mRNA expression analysis, flow cytometry to characterize membrane specific markers and ELISA to quantify secretion of cytokines/chemokines.

The data are informative but are not enough ambitious.

Determine the best time of polarization is necessary and the authors did it but I suggest adding more markers per polarization profile. In fact, it will be interesting to have the data for the same markers at the mRNA and secreted markers ; It was done only for TNFa in M1 MDM.

Moreover, some markers are not so specific to a type of polarization (CD206 for example) that is why it will be informative to have the expression in both the 3 phenotypes. In fact, to characterize a population of MDM from patients for example, we need to be confident with the difference of expression between subtypes.

The authors should modulate their conclusion, because the most important thing is to know if a marker is up or not and not so much how fold increased it is, except if we compared it to an another polarization profile ; that was not done here. In fcat, is more important to known when the marker is not expressed than is significantly expressed but lower.

I suggest to precise the % of viability cells after 24h, 48h and 72H, as LPS at 100 ng/ml may be strong for M1 MDM. These data can be extracted form flow cytometry analysis since a fixable viability kit was used. The pic of isotype control should appear on flow cytometry graph.

Student’t test cannot be used for the figures but an Anova test.

Please corrected page 10, paragraph 3.3 : “analyzed by flow cytometry” replace by “analyzed by ELISA”.

Please precise the number of replicate, it is not clear : if data are representative of 2 experiments, statistics cannot be done.
