# Supplementary material for: Effect of stimulation time on the expression of human macrophage polarization markers
Source: PLoS One. 2022 Mar 14;17(3):e0265196. doi: 10.1371/journal.pone.0265196 (PMC8920204; doi:10.1371/journal.pone.0265196)
Supplement: Attachment — Submitted filename: 20220224_Response to reviewers.docx [file pone.0265196.s010.docx]

**Response to the Reviewers, *PLOS ONE***

We were pleased to find that the reviewers and the editors thought that our manuscript entitled “Effect of stimulation time on the expression of human macrophage polarization markers” by Purcu *et al.,* was of interest and that we addressed all of their concerns raised in our revised manuscript. We thank you for giving us the opportunity to submit a revised version. We also appreciate the editor’s and the reviewers' time and effort in providing valuable feedback that has helped us to enhance the manuscript.

Please find the revised manuscript attached, which incorporates the reviewers’ minor comments and highlights the significant changes in the text in yellow. Please find our point-by-point response to the comments below.

We believe that we have adequately addressed all the concerns raised by the reviewers, and hope that our revised manuscript is now suitable for publication in *PLOS ONE*.

**Reviewer #2:**

**1- I suggest to add in the table 1 the new data on Il-10 and on TGF-b mRNA levels in M2a and M2c macrophages respectively**

**Response 1**

We thank the reviewer for this comment. We added information on IL-10 in M2a macrophages and TGFβ in M2c macrophages at mRNA level to the revised Table 1.

**2- See the reference of Actin B primers lines 168 an 173.**

**Response 2**

We thank the reviewer for this comment and apologize for the confusion. The Actin B primers mentioned in lines 168 and 173 have different reference ID’s as they are primers from different manufacturers. In the first version of the manuscript, all the real time PCR experiments were performed using the RealTime ready Single Assays by Roche. However, as these assays were later discontinued, for the revision we had to use a different assay with a different reference ID.
